# Surgical treatment strategy in Warthin tumor of the parotid gland^☆^

**DOI:** 10.1016/j.bjorl.2018.04.004

**Published:** 2018-05-16

**Authors:** Dong Hoon Lee, Tae Mi Yoon, Joon Kyoo Lee, Sang Chul Lim

**Affiliations:** a Chonnam National University, Medical School, Department of Otolaryngology-Head and Neck Surgery, Hwasun, South Korea; b Chonnam National University, Hwasun Hospital, Hwasun, South Korea

**Keywords:** Warthin tumor, Parotid gland, Computed tomography, Fine-needle biopsy, Surgical procedures, Tumor de Warthin, Glândula parótida, Tomografia computadorizada, Biópsia por agulha fina, Procedimentos cirúrgicos

## Abstract

**Introduction:**

Warthin tumors are the second most common benign tumors of the parotid gland. We examined the clinical features of Warthin tumors in our hospital, and analyzed the consistency within the literatures.

**Objective:**

The aim of this study is to analyze the clinical features of Warthin tumors in our 10-year experience of 118 Warthin tumors undergoing surgery at a single institute.

**Methods:**

From December 2006 to December 2016, 110 patients who underwent surgical treatment for Warthin tumors were identified based on their medical records.

**Results:**

A total of 118 parotid gland operations were performed in 110 patients. Almost 90% of Warthin tumors were found in males, and average patient age was 66.1 ± 6.1 years. The prevalence of smoking history was 89.1% (98/110). Eight patients (7.3%) had bilateral Warthin tumors. Seventy-seven lesions (65.3%) were located in the parotid tail portion, followed by 34 lesions in the superficial lobe (28.8%) and 7 lesions in the deep lobe (5.9%).

**Conclusion:**

We determined the appropriate extent of surgery depending on the fine needle aspiration cytology and tumor location by computed tomography scans. Partial facial dysfunction after the operation was detected in 12 cases, and facial nerve function recovered within 3 months. Only one patient experienced a recurrence, and was disease free after the re-operation. We suggest that our treatment algorithm, depending on the location of tumors and the result of fine needle aspiration cytology, can be useful to determine the appropriate extent of surgery for Warthin tumors.

## Resumo

**Introdução:**

Os tumores de Warthin são os segundos tumores benignos mais comuns da glândula parótida. Avaliamos as características clínicas dos tumores de Warthin em nosso hospital e analisamos a consistência com a literatura.

**Objetivo:**

Analisar as características clínicas dos tumores de Warthin em nossa experiência de 10 anos de 118 tumores de Warthin submetidos a tratamento cirúrgico em um único instituto.

**Método:**

De dezembro de 2006 a dezembro de 2016, 110 pacientes que receberam tratamento cirúrgico para tumores de Warthin foram identificados com base em seus prontuários médicos.

**Resultados:**

Foram feitas 118 cirurgias na glândula parótida em 110 pacientes. Quase 90% dos tumores de Warthin foram encontrados em homens e a média da idade dos pacientes foi de 66,1 ± 6,1 anos. A prevalência de tabagismo foi de 89,1% (98/110). Oito pacientes (7,3%) tinham tumores de Warthin bilaterais na glândula parótida. Das lesões, 77 (65,3%) localizavam-se na porção da cauda da parótida, seguidas por 34 no lobo superficial (28,8%) e 7 no lobo profundo (5,9%).

**Conclusão:**

Determinamos a extensão apropriada da cirurgia de acordo com a punção aspirativa com agulha fina e localização do tumor por tomografia computadorizada. Disfunção facial parcial após a cirurgia foi detectada em 12 casos e a função do nervo facial foi recuperada em 3 meses. Apenas um paciente apresentou recidiva e ficou livre da doença após reoperação. Sugerimos que nosso algoritmo de tratamento, a depender da localização dos tumores e do resultado da PAAF, pode ser útil para determinar a extensão apropriada da cirurgia para os tumores de Warthin.

## Introduction

Warthin tumors are the second most common benign tumors of the parotid gland, accounting for about 15% of all parotid tumors.^1^, ^2^, ^3^, ^4^, ^5^ Warthin tumors typically present as an asymptomatic, slowly growing mass, usually located in the tail (inferior) portion of the parotid gland.^1^, ^2^, ^5^, ^6^ Warthin tumors occur more frequently in males, with a peak incidence in the 5th and 6th decades. These tumors have a tendency toward multiplicity and bilaterality, and have an association with smoking.^1^, ^2^, ^3^, ^4^, ^5^, ^6^ Recently, many reports have presented patients with clinical features that differ from the traditional tumors in some way, such as increasing incidence among females, higher rates of tumor multiplicity and bilaterality.^4^, ^5^, ^6^ In addition, Warthin tumors show regional, national, and racial differences.^5^

We examined the clinical features of Warthin tumors in patients treated in our hospital, and analyzed the consistency within the relevant literature. The aim of this study was to analyze the clinical features of Warthin tumors in our 10-year experience of 118 Warthin tumors undergoing surgery at a single institute.

## Methods

After obtaining approval from the Institutional Review Board of our Hospital, a retrospective review (CNUHH-2017-134) was performed to evaluate patients who received surgical treatment for Warthin tumor of the parotid gland at the Department of Otolaryngology-Head and Neck Surgery in the hospital from December 2006 to December 2016. One hundred ten patients who received surgical treatment for Warthin tumor of the parotid gland were identified based on their medical records. Clinical data of patients with Warthin tumor of the parotid gland were reviewed, including age, sex, underlying diseases, smoking or alcohol, location of tumors, symptoms, duration of symptoms, fine needle aspiration cytology (FNAC), surgical procedures, complications and recurrence.

All patients underwent computed tomography (CT) scanning before surgery to assess the extent of the lesions and to aid in treatment planning. All patients, except for five patients, underwent FNAC.

The type and extent of surgery was dependent on the location of tumors and the result of FNAC. All patients underwent a macroscopically complete oncologic resection. Partial parotidectomy, involving enucleation or removal of the inferior half of the superficial lobe, was performed if the Warthin tumor was located in the tail (inferior) portion of the parotid gland. Superficial parotidectomy was performed if the tumor was located in the superficial lobe. Total parotidectomy was performed if the tumor was located in the deep lobe.

Intraoperative facial nerve monitoring was typically used. Postoperative drainage was performed and it was maintained by aspiration. All cases of Warthin tumors were confirmed histopathologically. Fisher's exact test was used for statistical analysis using SPSS version 20.0. Statistical significance was defined as a *p*-value < 0.05.

## Results

A total of 118 parotid gland operations were performed in 110 patients. Clinical data of 118 parotid gland operations for Warthin tumor are summarized in Table 1. This group of 110 patients included 98 males (89.1%) and 12 females (10.9%). The age at the time of diagnosis ranged between 37 and 85 years, with a mean of 66.1 ± 6.1 years. Among these 110 patients, 54 patients (49.1%) had underlying diseases, such as hypertension (*n* = 44), diabetes (*n* = 21), chronic hepatitis B (*n* = 5), and asthma (*n* = 4). The prevalence of smoking history was 89.1% (98/110), and the period of pack-years ranged from 2.4 pack-years to 100 pack-years with a mean period of 33.4 ± 19.0 pack-years. The rate of alcohol consumption was 56.4% (62/110).

**Table 1:** Clinical data of 118 parotid gland operations for Warthin tumor.

Factors	Value
Age (year): mean ± SD (range)	66.1 ± 6.1 (37–85)
Sex (male/female)	98/12
Smoking (yes/no)	98/12
Period of pack-years (year): mean ± SD (range)	33.4 ± 19.0 (2.4–100)
Location (right/left/both)	64/54/8
Duration of symptom (month): mean ± SD (range)	20.2 ± 50.6 (0.3–480)
Size of tumor (cm): mean ± SD (range)	2.9 ± 1.0 (1–7.3)
Period of follow-up (month): mean ± SD (range)	50.3 ± 34.1 (1–120)
Recurrence	1 (0.01%)

SD, standard deviation.

Of the 118 lesions, 64 Warthin tumors (54.2%) were located in the right parotid gland, and 54 Warthin tumors (45.8%) were located in the left parotid gland. Eight patients (7.3%) had bilateral Warthin tumors. Most of the tumors (107/118, 90.7%) presented as a slowly enlarging mass within the parotid gland. The remaining eleven tumors were incidentally diagnosed by positron emission tomography-CT (PET-CT, *n* = 10) or CT (*n* = 1). The majority of the lesions were asymptomatic. The duration of symptoms ranged from 0.3 months to 480 months, with a mean duration of 20.2 ± 50.6 months. The size of Warthin tumors ranged from 1 cm to 7.3 cm, with a mean size of 2.9 ± 1.0 cm.

Among the 113 Warthin tumors which underwent FNAC, 68 lesions were diagnosed as Warthin tumors, but the remaining 45 lesions could not be diagnosed as Warthin tumors preoperatively (Table 2). FNAC had a diagnostic sensitivity of 60.2%, a diagnostic specificity of 0%, a positive predictive value of 100%, a negative predictive value of 0%, and an accuracy of 60.2% in diagnosing Warthin tumors. No specific complications were observed after FNAC.

**Table 2:** The results of preoperative fine-needle aspiration cytology associated with tumor location.

Warthin tumor location	Warthin tumor (*n* = 68)	Benign cystic lesion (*n* = 18)	Inflammation (*n* = 16)	Sialadenitis (*n* = 11)
Tail portion (*n* = 75)	49	12	12	2
Superficial lobe (*n* = 34)	19	6	4	5
Deep lobe (*n* = 4)				4

Seventy-seven lesions (65.3%) were located in the tail portion of the parotid gland, 34 lesions (28.8%) were located in the superficial lobe, and 7 lesions (5.9%) were located in the deep lobe. Among the 77 Warthin tumors in the tail portion of the parotid gland, 34 lesions were removed by superficial parotidectomy, and the remaining 43 lesions were removed by partial parotidectomy. All Warthin tumors in the superficial and deep lobes of the parotid gland underwent superficial parotidectomy and total parotidectomy, respectively (Table 3). The most common surgical procedure was superficial parotidectomy (*n* = 68, 57.6%), followed by partial parotidectomy (*n* = 43, 36.5%) and total parotidectomy (*n* = 7, 5.9%).

**Table 3:** Summary of the surgical methods associated with tumor location.

Warthin tumor location	Partial parotidectomy (*n* = 43)	Superficial parotidectomy (*n* = 68)	Total parotidectomy (*n* = 7)
Tail portion (*n* = 77)	43	34	
Superficial lobe (*n* = 34)		34	
Deep lobe (*n* = 7)			7

Partial facial dysfunction after the operation was detected in 12 cases, and facial nerve function recovered within 3 months. We analyzed the risk factors that can cause facial nerve dysfunction (Table 4). When Warthin tumors are located in the deep lobe, the risk of facial nerve dysfunction is significantly higher (*p* < 0.05). After the 118 surgical procedures, 5 cases developed other minor complications, such as temporary salivary fistula (*n* = 4) or postoperative hematoma (*n* = 1). All minor complications resolved uneventfully.

**Table 4:** Analysis of risk factors for facial nerve dysfunction.

		Facial nerve dysfunction		Univariate analysis	Multivariate analysis
		Yes	No		
Tumor location	Deep	3	4	*p* = 0.023	*p* = 0.008 (HR = 11.269)
	Superficial or tail	9	102		

Tumor size	<2.87 cm	3	56	*p* = 0.125	*p* = 0.0058 (HR = 0.241)
	>2.87 cm	9	50		

HR, hazard ratio.

The mean follow-up period after surgery was 50.3 ± 34.1 months, with a range from 1 to 120 months. Only one patient experienced a recurrence, and was disease free after the re-operation.

## Discussion

Previous studies have suggested that Warthin tumors occur more commonly in males older than 60 years.^1^, ^2^, ^3^, ^4^, ^5^, ^6^ In this study, almost 90% of Warthin tumors were found in males, and average patient age was 66.1 ± 6.1 years. We also found a strong association between smoking and Warthin tumors. The prevalence of smoking history was very high (89.1%), as in the previous reports.^1^, ^2^, ^3^, ^4^, ^5^, ^6^ Eight (7.3%) of the 110 patients had bilateral Warthin tumors.

The exact pathogenesis of Warthin tumors is unknown.^7^, ^8^, ^9^ The predominant hypothesis suggests that this lesion arises from the salivary gland tissue entrapped within parotid lymph nodes during embryogenesis. Carcinogens in smoke seem to be an important risk factor for the occurrence of Warthin tumors. Several studies have also shown the role of progesterone receptors in the etiology of Warthin tumors. There are few studies assessing the role of viruses in the pathogenesis of Warthin tumor.

Similar to previous reports, an asymptomatic slowly growing mass in the parotid gland was the presenting symptom in this study.^1^, ^2^, ^5^, ^6^ For diagnosis of Warthin tumors, we performed FNAC and CT scans. FNAC is a simple and effective method to diagnose a salivary gland tumor.^10^ In this study, almost all patients had undergone preoperative FNAC. The sensitivity and specificity for the diagnosis of Warthin tumor were not high. However, all results of FNAC could be helpful to rule out malignancy. In cystic parotid lesions, FNAC is difficult as the smears are often hypocellular or of poor quality, and do not lead to an accurate diagnosis.^11^ The reason for the low specificity may be the small number of samples. In this study, FNAC results of all deep lobe Warthin tumors were inaccurate. In small lesions or deep lobe tumors, ultrasound-guided FNAC may be more useful than traditional blind FNAC.

CT provides reliable information about the location of the tumor.^5^, ^9^, ^10^ In addition, recent developments in CT technology can help identify even the smallest Warthin tumors.^10^ CT scans of Warthin tumors showed enhanced, well-circumscribed lesions with cystic components and solid stroma.^5^, ^9^, ^10^ In this study, we could obtain the preoperative information and perform treatment planning by using CT scans and FNAC.

**Figure 1:**
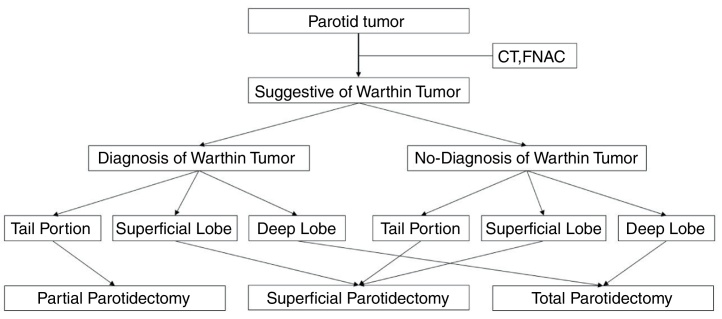
Algorithm for the surgical treatment of Warthin tumor of the parotid gland.

The treatment of choice for Warthin tumors is surgery.^5^, ^6^, ^9^, ^10^, ^11^, ^12^ However, there is a controversy about the appropriate extent of surgery.^5^, ^6^, ^12^ In this study, we determined the appropriate extent of surgery for Warthin tumors depending on the location of tumors and the result of FNAC (Fig. 1). In our hospital, when Warthin tumors were found, surgery was recommended. Periodic CT confirmation was performed on patients who refused surgery. According to this algorithm, 118 parotid gland surgeries were performed without recurrence except in one case. One patient with recurrence underwent superficial parotidectomy at the 1st surgery. However, the tumor recurred in the same region 9 months later, and it was removed by partial parotidectomy.

In the literature, the incidence of transient facial nerve dysfunction has been reported to range from 8% to 68% in all cases.^5^, ^6^, ^12^ In this study, the incidence of postoperative transient facial nerve dysfunction was 10.2% (12/118). All cases developed facial nerve dysfunction after superficial parotidectomy (*n* = 9) and total parotidectomy (*n* = 3). In this study, the risk of facial nerve dysfunction was significantly higher in Warthin tumors in the deep lobe than in superficial lobe or tail portion (*p* < 0.05). Other minor complications, including salivary fistula and hematoma, occurred after only superficial parotidectomy. Both facial dysfunction and minor complications resolved uneventfully.

The limitations of this study are the small sample size and a retrospective review. A prospective study including a large sample size is necessary to establish our treatment algorithm for Warthin tumors.

## Conclusion

We demonstrated that Warthin tumors occurred more frequently in elderly males with a tendency toward bilaterality, and an association with smoking. Our treatment algorithm, depending on the location of tumors and the result of FNAC, can be useful to determine the appropriate extent of surgery for Warthin tumors.

## Conflicts of interest

The authors declare no conflicts of interest.
